# Long non-coding RNA TINCR promotes hepatocellular carcinoma proliferation and invasion via STAT3 signaling by direct interacting with T-cell protein tyrosine phosphatase (TCPTP)

**DOI:** 10.1080/21655979.2021.1930336

**Published:** 2021-05-30

**Authors:** Chengwu Tang, Wenming Feng, Ying Bao, Huimin Du

**Affiliations:** a Department of General Surgery, The First People’s Hospital Affiliated to Huzhou Normal College, Huzhou, Zhejiang, People’s Republic of China; b Out-Patient Department, The First People’s Hospital Affiliated to Huzhou Normal College, Huzhou, Zhejiang, People’s Republic of China

**Keywords:** Hepatocellular carcinoma, RNA pull down, TINCR, TCPTP, STAT3

## Abstract

The long non-coding RNAs (lncRNAs) participate in modulating numerous important cancer phenotypes via formation of RNA-protein complex. TINCR (terminal differentiation-induced lncRNA) modulates cancer cell behavior in many human malignancies, such as hepatocellular carcinoma (HCC). Herein, we proposed to investigate the underlying mechanism by which TINCR regulates HCC progression via formation of RNA-protein. RNA pulldown, LC-MS/MS, bioinformatics analysis, and RNA immunoprecipitation (RIP) assays were employed to identify TINCR-interacting protein TCPTP in HCC cells. The siRNAs for TINCR and TCPTP were transfected into HCC cells. The plasmids encoding full length or the 1–360 nt deletion of TINCR were generated and applied to cell transfection. The CCK-8, colony formation, EdU, wound healing along with transwell assays were employed to examine cell proliferation, apoptosis, migration, and infiltration. Real-time PCR, as well as western blot assays were employed to assess the levels of STAT3 phosphorylation and its target genes. We identified 1–360 nt region of TINCR, which directly bound with the phosphatase domain of TCPTP to inhibit its tyrosine phosphatase activity. Then, the results showed that the increasing of cell growth, migration, infiltration, and the reducing of apoptosis in TINCR-knockdown HCC cells was remarkably reversed with TCPTP silence. Additionally, Δ1-360 TINCR overexpression did not affect HCC cell growth, apoptosis, migration, infiltration, and STAT3 target genes expression. Our data revealed that TINCR directly bound TCPTP and suppressed the dephosphorylation of STAT3, thus promoting STAT3 activation and its downstream target genes in HCC progression and tumorigenicity.

Highlights

LncRNA TINCR interacted with protein TCPTP

LncRNA TINCR maintained STAT3 phosphorylation

LncRNA TINCR affected STAT3 signaling in HCC

Abbreviations:

lncRNAs: long non-coding RNAs; TINCR: terminal differentiation-induced lncRNA; TCPTP: T cell protein tyrosine phosphatase; siRNA: small-interfering RNA; HCC: hepatocellular carcinoma; nt: nucleotide; LC-MS/MS: Liquid Chromatography - Tandem Mass Spectrometry; RIP: RNA immunoprecipitation; ANOVA: analysis of variance; EdU: 5-ethynyl-2’-deoxyuridine; real-time PCR: real-time polymerase chain reaction; CCK-8: cell counting kit-8; aa: amino acids; STAT3: signal transducer and activator of transcription 3

## Graphical abstract

**Figure:**
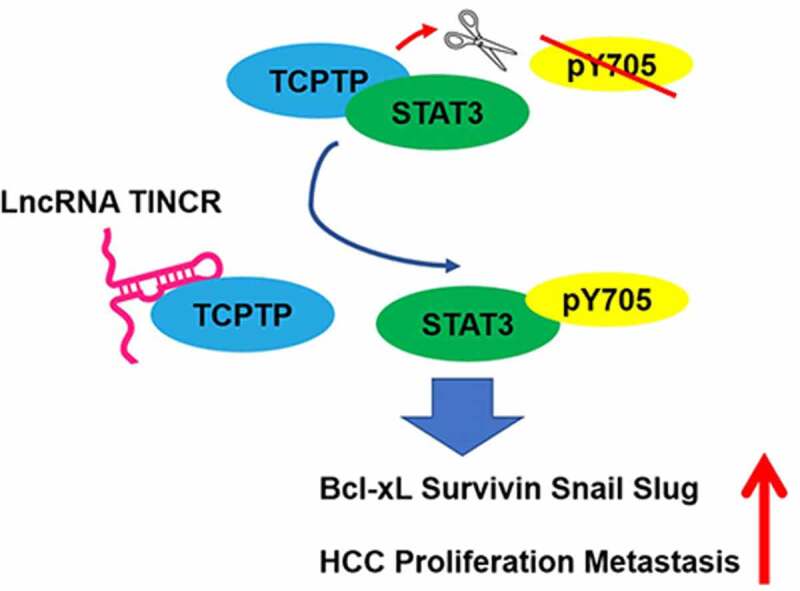

## Introduction

The long non-coding RNAs (lncRNAs) play critical roles in cell signaling cascades by various mechanisms, including the formation of protein-protein or RNA-protein complexes and modulation of protein modification, translation, and transcription [1]. Alterations of lncRNA expression have been found in numerous kinds of cancer by many genome-wide association studies and involved in various important cancer phenotypes [2,3]. Hepatocellular carcinoma (HCC) is one kind of human malignancies with leading lethal malignancy worldwide. Numerous studies have discovered that tumor-specific lncRNAs are promising novel HCC treatment targets, as well as prognostic biomarkers [4–8]. Therefore, the understanding of molecular mechanisms underlying liver carcinogenesis of lncRNA remain emergency.

LncRNA TINCR (terminal differentiation-induced lncRNA) has a long non-coding RNA (lncRNA) generating a 3.7-kb transcript. TINCR interacted with staufen1 (STAU1) protein and accelerated the progress of gastric cancer through activating the STAU1-CDKN2B signaling cascade [9,10]. TINCR promoted epithelial-mesenchymal transition through targeting microRNA-125b in mammary carcinoma [11]. Furthermore, LncRNA TINCR modulates cancer cell behavior in diverse kinds of cancer, for example HCC [12–14]. TINCR regulated HCC cell proliferation and invasion via targeting the miR-218-5p/DEAD-box helicase 5 (DDX5) and miR-214-5p/ROCK1 axes [15–17].

The aim of our study is to demonstrate the binding protein of TINCR and the mechanism by which TINCR regulates HCC progression via formation of RNA-protein. Here, we employed RNA pull down along with mass spectrometry strategies to determine the interaction proteins of lncRNA TINCR. Using these methods, we successfully characterize the TINCR interacting protein, the nuclear isoform of TCPTP (T cell protein tyrosine phosphatase, TC45), which interacts with STAT3 and dephosphorylates STAT3 Y705 [18]. Furthermore, STAT3 is always activated and promotes tumor aggressiveness in human HCC [19,20]. Bioinformatics analyses were employed to further explore the mass spectrometry data and the cross-talk regions between TINCR and TCPTP. In summary, we demonstrated that TINCR preserved STAT3 phosphorylation via direct interacting with TCPTP, thus activating STAT3 downstream target genes and promote HCC cell growth, migration, and infiltration.

## Materials and methods

### Cell culture

Hep3B and HCCLM3 cells were supplied by Beyotime (Shanghai, China). All cell lines were evaluated for Mycoplasma and characterized by STR profiling. The cells were grown in DMEM medium enriched with 10% FBS and 1% streptomycin/penicillin/(100 g/mL) under 37°C and 5% CO_2_ conditions.

### Plasmids and transfection

The full length (WT, 1–3733 nt) and Δ1-360 (360–3733 nt) of TINCR (NR_027064.3) were obtained by gene synthesis from GENEWIZ (Suzhou, China) and cloned into the pcDNA3 vector. The empty vector and pcDNA3-WT or pcDNA3-Δ1-360 plasmids were inserted into Hep3B or HCCLM3 cells via transfection with Lipofectamine 3000 (Thermo Scientific, Madison, USA) as described in manufacturer’s manual. The small interfering RNA (siRNA) duplexes against human TINCR were provided by GenePharma (Shanghai, China) (sequences as shown in Table 1). The siRNA duplexes against human TCPTP (#sc-76635) was supplied by Santa Cruz Biotechnology (Santa Cruz, USA). The siRNAs duplexes were inserted into Hep3B or HCCLM3 cells via transfection with Lipofectamine 3000 as described by the manufacturer.

**Table 1.** Primers and siRNAs used in this study

Primers and siRNAs	Sequence (5ʹ-3ʹ)
As-f	TAATACGACTCACTATAGGG TTGTTTTCAAACATGTAATC
As-r	GGGCGGGCGGAGCGCGGGCG
FL-f	TAATACGACTCACTATAGGGGGGCGGGCGGAGCGCGGGCG
D1-f	TAATACGACTCACTATAGGG AGCGACCCCAGGTAGTCTGG
D2-f	TAATACGACTCACTATAGGG AGGCCTCCAACTGTGCCCCA
D3-f	TAATACGACTCACTATAGGG GCTTTGCAGAATGACTTGGG
R1	TTGTTTTCAAACATGTAATC
D4-f	TAATACGACTCACTATAGGGGGGCGGGCGGAGCGCGGGCG
D4-r	CAGCTCCAGCAGGTCTGCCT
RIP-f	CTGCTACCGCTGACCGTG
RIP-r	GCCGCGCGTTGTAGTAGAAG
TINCR-f	CCAAGGAGGTTGTCAGGGAC
TINCR-r	TAGATACACGCATGTGGCCC
TCPTP-f	GAAGAGTTGGATACTCAGCGTC
TCPTP-r	TGCAGTTTAACACGACTGTGAT
Bcl-xL-f	GAGCTGGTGGTTGACTTTCTC
Bcl-xL-r	TCCATCTCCGATTCAGTCCCT
Cyclin D1-f	GCTGCGAAGTGGAAACCATC
Cyclin D1-r	CCTCCTTCTGCACACATTTGAA
Survivin-f	AGGACCACCGCATCTCTACAT
Survivin-r	AAGTCTGGCTCGTTCTCAGTG
Snail-f	TCGGAAGCCTAACTACAGCGA
Snail-r	AGATGAGCATTGGCAGCGAG
Slug-f	TGTGACAAGGAATATGTGAGCC
Slug-r	TGAGCCCTCAGATTTGACCTG
β-actin-f	CATGTACGTTGCTATCCAGGC
β-actin-r	CTCCTTAATGTCACGCACGAT
TINCR siRNA-1	1523-GAAATAATGTTTTAGTTAAGA
TINCR siRNA-2	2160-GAAAATGGGGCATTTAATAAT
TINCR siRNA-3	3680-CAGAAATGCTGTTTTGAGAGT

### RNA pull down assay

RIP was conducted as documented in previous study [21]. For *in vitro* transcription of TINCR fragment constructions (Antisense 1–3733 nt, FL 1–3733 nt, D1 360–3733 nt, D2 1100–3733 nt, D3 1800–3733 nt, D4 1–1800 nt), primers containing T7 promoter sequence (TAATACGACTCACTATAGGG) and PCR methods were used to obtain the templates. The primers were purchased from GENEWIZ (Suzhou, China) (primer sequences as indicated in Table 1). The PCR products were assessed via DNA agarose gel electrophoresis (0.5%) and purified with Quick Gel Extraction Kit (Transgen, Beijing, China). Biotin-conjugated sense along with antisense chains of TINCR fragment constructions were transcribed *in vitro* with a Biotin RNA Labeling Mix (#11685597910, Roche, Basel, Switzerland), as well as a T7 High Efficiency Transcription Kit (Transgen) as described by the manufacturer. The synthesized RNAs were examined with Denaturing Gel Buffer (#AM8676, Thermo Scientific) formaldehyde-containing agarose gels (1%) as documented in the manual. The synthesized RNAs were purified using an RNA Purification Kit (Transgen) and detected by dot blot with HRP-Streptavidin (Beyotime) and BeyoECL Moon (Beyotime) as documented in the manufacturer provided manual. For RNA pull down assay, RIPA lysis buffer (Beyotime) enriched with protease inhibitor cocktail (Beyotime) and 1 mM PMSF (Beyotime) was employed to lyse the Hep3B cells. Afterward, the BCA Protein Assay Kit (Beyotime) was employed to quantify the proteins. Then, we mixed 1 mg of Hep3B cellular proteins with different biotinylated TINCR RNA, and 1 μm Streptavidin Dynabeads (#MB1003, Nanoeast, Nanjing, China) were introduced and incubated at room temperature (RT) for 2 h. Next, the beads were rinsed and boiled for 20 minutes with 1 X SDS-PAGE sample loading buffer. Then, the interacting proteins were purified by Magnetic Separation Rack (Beyotime) and were applied to SDS-PAGE, western blot, and silver staining.

### Silver staining and mass spectrometry assays

The purified proteins were fractionated via SDS-PAGE with Resolving Gel Master Mix (Beyotime) and were visualized using silver staining by Fast Silver Stain Kit (Beyotime) as described by the manufacturer. The target bands were removed and digested by Sequencing Grade Modified Recombinant Trypsin (Beyotime). The mass spectrometry assay was conducted by Servicebio (Wuhan, China). Raw data were searched against the Uniprot human protein data resource.

### Bioinformatics analysis

The interaction fragments between LncRNA TINCR (NR_027064.3) and protein TCPTP (isoform 2, NP_536347) were predicted by using catRAPID omics algorithm (Methods: Fragments, http://service.tartaglialab.com/page/catrapid_group) [22]. The 3D structure of protein TCPTP was obtained from Protein Data Bank (PDB entry: 1L8K, https://www.ebi.ac.uk/pdbe/entry/pdb/1L8K). The secondary structure of LncRNA TINCR was predicted by using the mfold web resource (http://www.bioinfo.rpi.edu/applications/mfold) [23].

### RNA immunoprecipitation (RIP) assay

RIP was done as documented previously [24]. Concisely, cells were inoculated with Trypsin (Beyotime) and washed with PBS, then crosslinked in 1% formaldehyde for 10 minutes at RT. After that, the cells were inoculated with 2.5 M glycine for five minutes at RT to block formaldehyde. Next, we re-suspended the cell pellet in RIPA buffer (Beyotime) enriched with RNasin (Tiangen, Beijing, China), protease inhibitor cocktail (Beyotime), and 1 mM PMSF (Beyotime). The cell suspension was briefly sonicated and span for three minutes at 14,000 × g at RT. Normal Rabbit IgG (#2729, CST, Beverly, USA) or TCPTP (#58935, CST) antibodies were pre-bound with Protein G Sepharose beads for 3 hours at RT. Thereafter, protein G Sepharose beads (Abcam, Cambridge, UK) were employed to pre-clear the supernatant and then introduced to the beads and incubated on a rotating wheel overnight at 4°C. Beads were then rinsed with RIPA buffer. We reversed the crosslinks and Proteinase K (Beyotime) was employed to digest the proteins at 65°C for two hours. After that, RNA was extracted by using TRNzol Universal (Tiangen) and reverse-transcripted by using FastKing-RT SuperMix (Tiangen) as documented in the manual provided by the manufacturer.

### RT-qPCR

The cellular RNA was extracted by using TRNzol Universal (Tiangen) and reverse-transcripted by using FastKing-RT SuperMix (Tiangen) as described by the manufacturer. The real-time PCR was run on a 7500 Real-Time PCR System (ABI, Foster City, CA, USA) using FastFire SYBR Green qPCR PreMix (Tiangen). The primers for RIP and mRNA expression were purchased from GENEWIZ (Suzhou, China) (sequences as indicated in Table 1) and β-actin was employed as the normalization standard.

### Western blotting

The RIPA lysis buffer (Beyotime) enriched with protease inhibitor cocktail (Beyotime), and 1 mM PMSF (Beyotime) was employed to isolate the cellular proteins. Subsequently, the BCA Protein Assay Kit (Beyotime) was employed to quantify the proteins as documented in the manual provided by the manufacturer. The proteins were fractionated in a SDS-PAGE gel with Resolving Gel Master Mix (Beyotime) and blotted onto PVDF membrane (Beyotime). The membranes were blocked with Blocking Buffer (Beyotime) and incubated with primary antibodies (TCPTP, #58935; Stat3, #9139; p-Stat3, #9145, CST; Bcl-xL, AB126; Cyclin D1, AF1183; Survivin, AF1222; Snail, AF8013; E-Cadherin, AF0138; Vimentin, AF0318; Slug, AF7998; β-actin, AF5003, Beyotime) at 4◦C overnight and secondary antibodies, that is, HRP-conjugated Goat Anti-Mouse IgG, A0216; HRP-conjugated Goat Anti-Rabbit IgG, A0208, Beyotime) at RT for 2 hours. The blots were visualized using BeyoECL Moon (Beyotime) as described by the manufacturer. β-Actin served as the internal standard.

### CCK-8 assay

Cell proliferation was assayed by using the enhanced cell counting kit-8 (CCK-8) assay (Beyotime) as documented in the manual provided by the manufacturer. Concisely, 2 × 10^3^ cells/100 μL/well were inoculated in a 96-well plate and allowed to grow for 24, 48 or 72 h, respectively. 10 μL of CCK-8 solution was introduced to every well and incubated under 37°C and 5% CO_2_ conditions for 1 h before the absorbance was determined at 450 nm.

### Colony formation

500 cells/1 mL/well were inoculated in a six-well plate. The medium was replaced every 4 days and grown for 16 days. Then, the cells were fixed with methanol and visualized by using Crystal Violet Staining Solution (Beyotime) as described by the manufacturer.

### EdU assay

The EdU assay was conducted by using EdU Cell Proliferation Kit with Alexa Fluor 488 (Beyotime) as described by the manufacturer. Briefly, cells were planted at 2 × 10^4^ cells/1 mL/well in a six-well plate and incubated under 37°C and 5% CO_2_ conditions for 12 hours. Then, 1 mL EdU solution (20 μM) was introduced to every well and incubated at 37°C for 4 h. Next, the cells were fixed with methanol and washed with PBS. Next, 0.5 mL Click Reaction Mix was introduced to every well and incubated at RT for 30 minutes. The cells were counter stained with DAPI Staining Solution (Beyotime) and examined using Olympus IX73 inverted fluorescent microscope system (Olympus, Tokyo, Japan).

### Apoptosis assay

The Annexin V-FITC Apoptosis Detection Kit (Beyotime) was employed to explore cell apoptosis as documented in the manufacturer’s manual. Concisely, 5 × 10^4^ cells/2 mL/well were inoculated in a six-well plate and incubated under 37°C and 5% CO_2_ conditions for 24 hours. Then, cells were collected with Trypsin (Beyotime) and rinsed with PBS. Next, 195 μL Annexin V-FITC binding buffer, 5 μL Annexin V-FITC, along with 10 μL Propidium Iodide were introduced to every sample and incubated at RT for 20 minutes. The samples were assessed with a BD FACSCalibur flow cytometer (BD Biosciences, San Jose, USA) and analyzed by the BD FlowJo software.

### Wound healing assay

10^5^ cells/2 mL/well were planted in a six-well plate and incubated under 37°C and 5% CO_2_ conditions for 24 hours. Then, the cell monolayer was scratched using p200 pipet tip and rinsed with PBS. Thereafter, the cells were inoculated in DMEM medium enriched with 2% FBS for 48 hours under 37°C and 5% CO_2_ conditions. The wound distance was observed with a microscope (Olympus).

### Transwell assay

The Matrigel (Corning, NY, USA) was diluted with serum-free DMEM medium and inoculated into the Invasion Compartment with 8.0 µm PET Membrane (Corning). Afterward, 10^5^ cells/100 μL/well were inoculated in the upper compartment and the DMEM enriched with 20% FBS was introduced into lower well of a 24-well plate. Then, the cells were incubated under 5% CO_2_ and 37°C conditions for 24 hours. The upper chamber was rinsed with PBS, fixed with methanol, followed by Crystal Violet Staining. The number of invasion cells was counted with a microscope (Olympus).

### Statistical analysis

The data are shown as mean and standard error of mean (SEM) from three independent experiments. All statistical analyses were implemented in the GraphPad Prism V.5 (GraphPad Software, CA, USA). The data were analyzed by Student’s *t* tests between two groups, whereas analysis of variance (ANOVA) among multiple groups. The Mann–Whitney *U* test was employed when the data were not normally distributed. *, *P* < 0.05 signified statistical significance.

## Results

### LncRNA TINCR interacts with protein TCPTP

**Figure 1.**
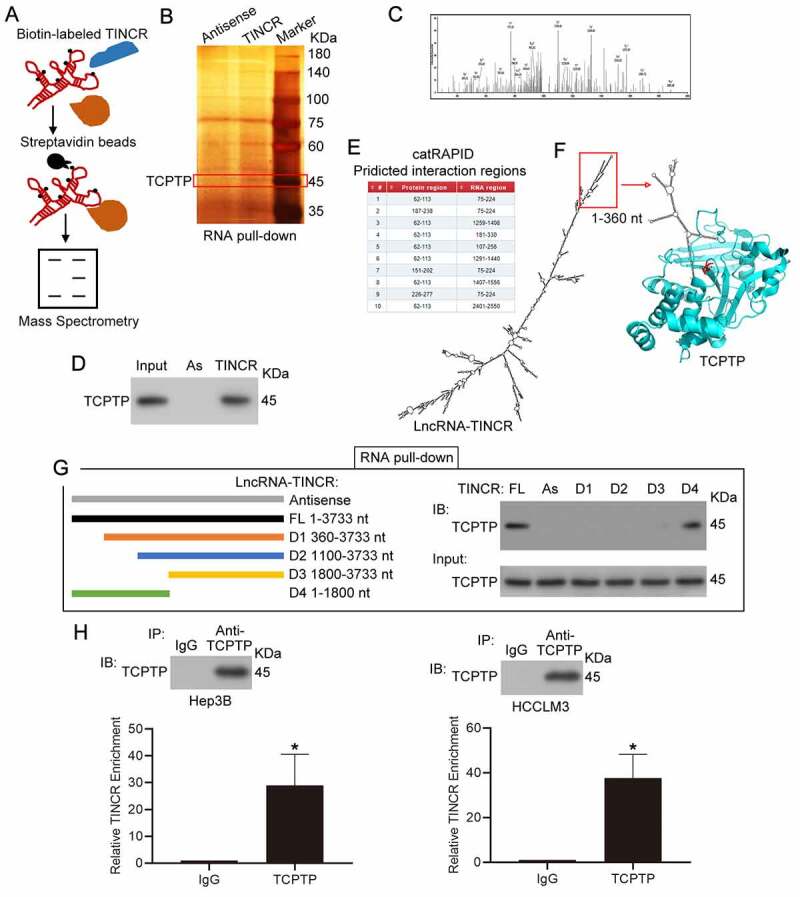
**LncRNA TINCR correlated with TCPTP protein**. (a) Schematic diagram of RNA pull-down assay/mass spectrometry procedures. (b) RNA pull-down assay was conducted by using antisense synthesized TINCR RNA (Antisense) or full-length sense synthesized TINCR RNA (TINCR). The purified interacting proteins from Hep3B was detected by using SDS-PAGE and silver staining. Red box indicates position of gel slice for mass spectrometry. (c) Annotated MS/MS spectrum assigned to the TCPTP peptide CAQYWPTDDQEMLFK from TINCR vs. TINCR antisense RNA pull-down assay. (d) Western blot experiment demonstrated the interaction between TCPTP and TINCR. The purified interacting proteins from Hep3B was obtained from TINCR vs. TINCR antisense RNA pull-down assay. AS, TINCR antisense RNA. (e) The interaction fragments between LncRNA TINCR (NR_027064.3) and protein TCPTP (isoform 2, NP_536347) were predicted by using catRAPID omics algorithm. (f) Visualization of interaction between TCPTP and TINCR was simulated by Protein Data Bank and mfold web server. The active site (cysteine 216) of TCPTP is shown in red. (g) Schematic diagram for generating *in vitro* transcription of biotinylated TINCR fragment constructions (Antisense 1–3733 nt, FL 1–3733 nt, D1 360–3733 nt, D2 1100–3733 nt, D3 1800–3733 nt, D4 1–1800 nt). The Hep3B cellular protein was extracted for RNA pull down assay. The different TINCR *in vitro* transcriptions were employed to perform RNA pull-down assay, and western blotting was performed to detect TCPTP protein in purified proteins and Input. (h) Western blot for TCPTP and real-time PCR for TINCR using RNA immunoprecipitation (RIP) enriched samples of Hep3B and HCCLM3 cells. Data are given as mean ± SEM (n = 3, *P < 0.05, by Student’s t-test)

To characterize the cross-talking proteins of lncRNA TINCR, an approach was used by combining RNA pull-down, SDS-PAGE fractionation and LC-MS/MS assays. As indicated in Figure 1(a), we first synthesized biotinylated sense and antisense TINCR with *in vitro* transcription (Table 1) for RNA pull down assay. Biotin-UTP was incorporated randomly into the TINCR RNA during transcription. The biotinylated sense and antisense TINCR was inoculated with cellular proteins isolated from Hep3B cells. The interacting proteins were purified by streptavidin beads and were applied to SDS-PAGE and silver staining. The target band (~45KDa) was removed and examined by LC-MS/MS (Figure 1(b) and Supplementary Table 1). Apart from several RNA binding proteins, we found that TINCR might interact with protein TCPTP, a kind of non-receptor type tyrosine-specific phosphatase (Figure 1(c)). To verify the MS result, western blot experiment was employed to detect TCPTP from the purified interacting proteins from Hep3B, and the results confirmed that TINCR interacted with protein TCPTP (Figure 1(d)). To explore the interaction regions between lncRNA TINCR (NR_027064.3) and protein TCPTP (isoform 2, NP_536347), we performed bioinformatics analysis by catRAPID omics algorithm (Figure 1(e)) and simulated the interaction between TCPTP and TINCR by Protein Data Bank and mfold web server (Figure 1(f)). The 75–224 nt region of TINCR might interact with aa 62–113 and aa 187–238 regions of TCPTP, thus suggesting that TINCR could competitive bind TCPTP catalytic cysteine residue (cysteine 216) and inhibit its tyrosine phosphatase activity. Additionally, 1–360 nt region of TINCR forms an independent secondary structure (Figure 1(f)). Based on the bioinformatics analysis, different *in vitro* transcription of TINCR fragments (Antisense, FL 1–3733 nt, D1 360–3733 nt, D2 1100–3733 nt, D3 1800–3733 nt, D4 1–1800 nt) were applied to RNA pull down analysis, and the purified interacting proteins from Hep3B was assessed by SDS-PAGE along with western blot assays. The FL and D4 of TINCR could interact with TCPTP, but not D1, D2, and D3 (Figure 1(g)). These results suggested that 1–360 nt region of TNICR is responsible for interacting with TCPTP. Next, the levels of TINCR and TCPTP in hepatoma cell lines were measured, and the results showed that the expression of TINCR was higher in Hep3B and HCCLM3 cells than others (Supplementary Figure 1(a)). However, the expression of TCPTP was no difference in hepatoma cell lines (Supplementary Figure 1(b)). To confirm the interaction of TNICR and TCPTP, RIP experiment and real-time PCR assay were performed in Hep3B and HCCLM3 cells. Then, we observed remarkable enrichment of TINCR in the immunoprecipitates of TCPTP in contrast with the normal IgG control (Figure 1(h)). Overall, these results suggested that the 1–360 nt region of TINCR was required for its direct interaction with TCPTP in both Hep3B and HCCLM3 HCC cells.

### TINCR modulates HCC cell growth and apoptosis through TCPTP

**Figure 2.**
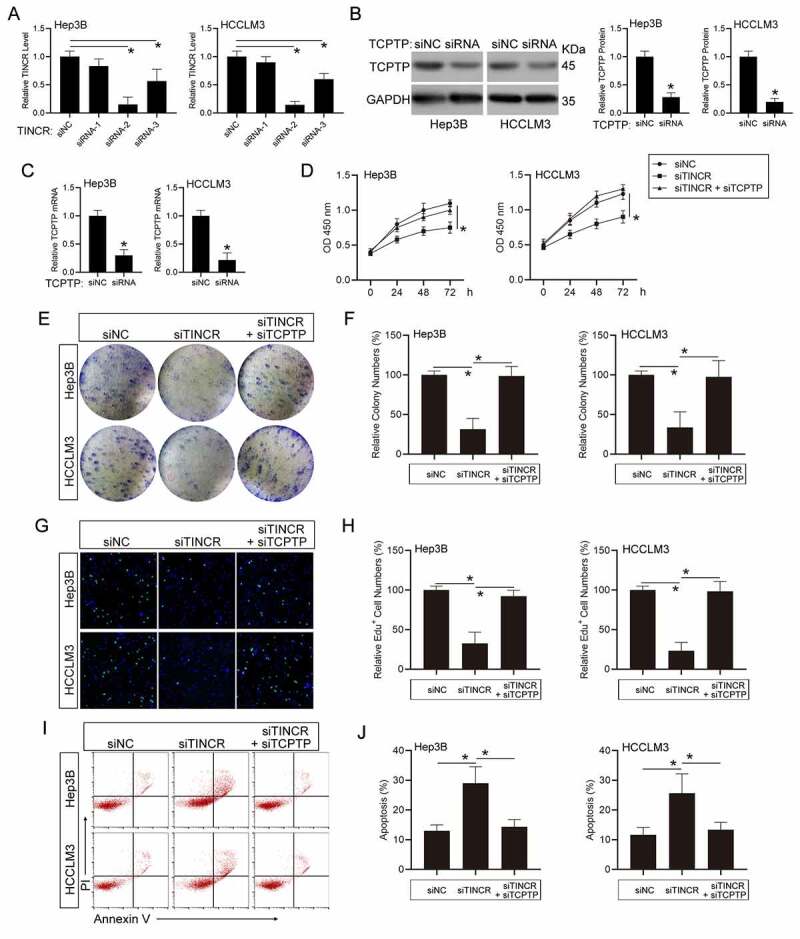
**LncRNA TINCR promotes hepatocellular carcinoma (HCC) cell growth and inhibits apoptosis through TCPTP**. (a) Real-time PCR assay of TINCR expression in different TINCR siRNA-silenced Hep3B and HCCLM3 cells. Data are indicated as mean ± SEM (n = 3, *P < 0.05, by ANOVA). (b) Western blot assay of TCPTP in TCPTP siRNA-silenced Hep3B and HCCLM3 cells. Data are indicated as mean ± SEM (n = 3, *P < 0.05, by Student’s t-test). (c) Real-time PCR assay of TCPTP in TCPTP siRNA-silenced Hep3B and HCCLM3 cells. Data are shown as mean ± SEM (n = 3, *P < 0.05, by Student’s t-test). The CCK-8 (d), colony formation (e and f), EdU (g and h), and apoptosis (i and j) assays were performed in Hep3B and HCCLM3 cells transfected with siRNA-NC, siRNA-TINCR, or co-transfected with siRNA-TINCR and siRNA-TCPTP. Data are given as mean ± SEM (n = 3, *P < 0.05, by ANOVA)

To explore whether TINCR plays its roles in modulating HCC cell growth along with apoptosis through TCPTP, we silenced TCPTP expression in TINCR-knockdown HCC cells. Firstly, we designed three TINCR-target siRNAs and transfected into Hep3B and HCCLM3 HCC cells. The results showed that siRNA-2 remarkably inhibited TINCR expression, as well as siRNA-3 (Figure 2(a)). Hence, TINCR siRNA-2 was utilized in the following experiments. Additionally, the expression of both TCPTP protein and mRNA levels was decreased in TCPTP siRNA transfected HCC cells (Figure 2(b,c)). To observe the influence of TINCR on cell growth in HCC, the CCK-8 assay was performed in co-insertion of TINCR-siRNA and TCPTP-siRNA HCC cells via transfection. The data illustrated that TINCR silenced-induced cell growth inhibition was reversed by TCPTP silence in Hep3B and HCCLM3 HCC cells (Figure 2(d)). The colony-formation assay similarly illustrated that TINCR silencing reduced cell growth. However, co-transfection of TINCR-siRNA and TCPTP-siRNA remarkably increased the cell growth that was inhibited by TINCR silence in Hep3B and HCCLM3 HCC cells (Figure 2(e,f)). To elucidate the mechanism through which TINCR modulates cell growth, we carried out EdU assay and the results illustrated that the ratio of EdU-positive cells was remarkably reduced after TINCR silencing. In addition, reduced ratio of EdU-positive cells in TINCR-knockdown group was remarkably increased with TCPTP-siRNA co-transfection (Figure 2(g,h)), indicating that TINCR had an important role in regulating Hep3B and HCCLM3 HCC cells S phase of the cell cycle. Next, apoptosis assay illustrated that silencing of TINCR remarkably induced cell apoptosis in contrast with the control in Hep3B and HCCLM3 HCC cells. By contrast, silence of TCPTP in TINCR-silenced HCC cells repressed cell apoptosis (Figure 2(i,j)). Altogether, these data suggest that TINCR enhances cell growth and represses apoptosis through TCPTP.

### TINCR regulates HCC cell migration and infiltration through TCPTP

**Figure 3.**
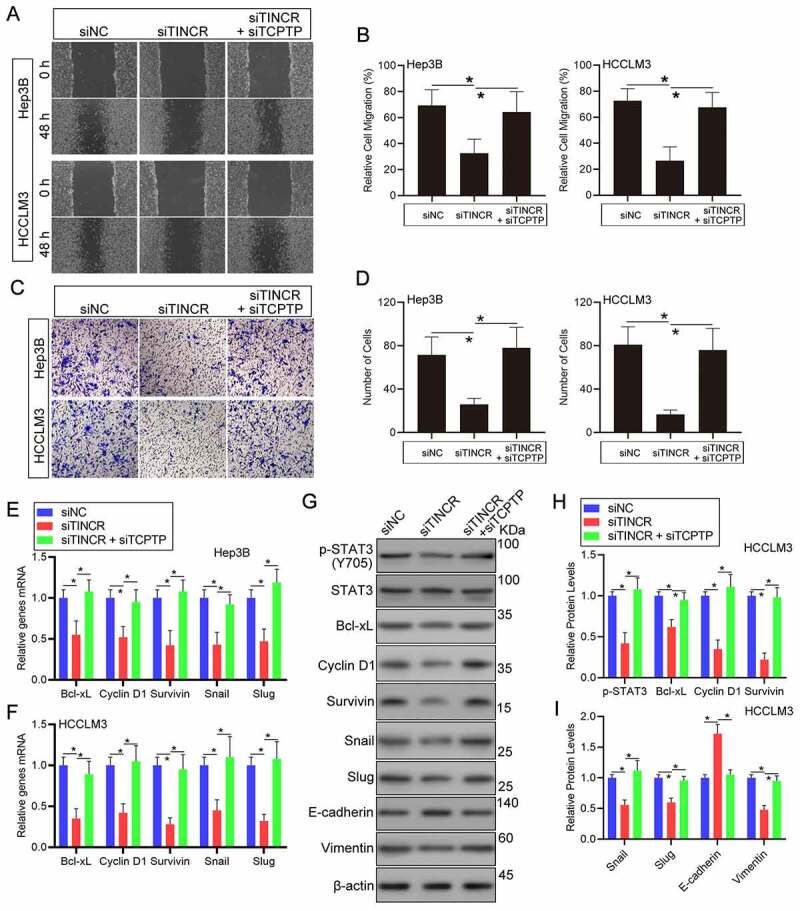
**LncRNA TINCR promotes HCC cell invasion and STAT3 signaling through TCPTP**. The wound healing (a and b) and transwell (c and d) assays were conducted in Hep3B and HCCLM3 cells transfected with siRNA-NC, siRNA-TINCR, or co-transfected with siRNA-TINCR and siRNA-TCPTP. Data are given as mean ± SEM (n = 3, *P < 0.05, by ANOVA). (e and f) Real-time PCR assay of STAT3 target genes expression in Hep3B and HCCLM3 cells transfected with siRNA-NC, siRNA-TINCR, or co-transfected with siRNA-TINCR and siRNA-TCPTP. Data are indicated as mean ± SEM (n = 3, *P < 0.05, by ANOVA). **(G, H and I)** Western blot assay of STAT3 phosphorylation and STAT3 target genes in HCCLM3 cells transfected with siRNA-NC, siRNA-TINCR, or co-transfected with siRNA-TINCR and siRNA-TCPTP. Data are given as mean ± SEM (n = 3, *P < 0.05, by ANOVA)

To explore if TINCR affects HCC cell migration and infiltration through TCPTP, a set of experiments were carried out in HCC cells co-transfection with TINCR-siRNA and TCPTP-siRNA. Wound healing assay demonstrated that TINCR silence remarkably repressed cell migration in Hep3B and HCCLM3 HCC cells. But, silencing of TCPTP partially attenuated the decreased cell migration capacity caused by TINCR silence (Figure 3(a,b)). Similarly, the results of transwell assay illustrated that HCC cells invasion was remarkably decreased following silencing of TINCR. Then, the rescue experiments showed that TCPTP silence reversed the decrease in cell invasion capacity caused by TINCR silencing (Figure 3(c,d)). Collectively, these data suggest that TINCR enhances cell migration and infiltration through TCPTP.

### TINCR regulates the phosphorylation of STAT3 and its downstream target genes expression through TCPTP

The catalytic cysteine residue (cysteine 216) and its surrounding residues of TCPTP is responsible for its tyrosine phosphatase activity of TCPTP [20]. TCPTP can directly dephosphorylate STAT3 in mice models with NASH or HCC [19]. To verify that the catalytic cysteine residue of TCPTP can be competitive combined and the phosphatase activity can be inhibited by TINCR, the levels of STAT3 phosphorylation and its downstream target genes were performed in HCC cells co-transfection with TINCR-siRNA and TCPTP-siRNA. Then, we measured the expression of STAT3 downstream target genes. The results of real-time PCR assay revealed that Cyclin D1, Bcl-xL, Survivin, Snail, and Slug mRNA levels were remarkably decreased following silencing of TINCR in Hep3B and HCCLM3 HCC cells. Furthermore, silencing of TCPTP could increase these genes mRNA expression in TINCR silence HCC cells (Figure 3(e,f)). Indeed, the STAT3 phosphorylation level was decreased in TINCR-silenced HCC cells in contrast with the corresponding control cells. Moreover, the co-transfection of TINCR-siRNA and TCPTP-siRNA reversed the decrease in STAT3 phosphorylation in response to TCPTP silence, suggesting that TINCR regulated STAT3 phosphorylation through TCPTP (Figure 3(g–i)). Similarly, the results of western blot assay revealed that Cyclin D1, Bcl-xL, Survivin, Snail, and Slug protein levels were remarkably decreased following silencing of TINCR in HCCLM3 HCC cells. Then, the rescue experiments showed that TCPTP silence reversed the decrease of these protein levels caused by TINCR silencing (Figure 3(g–i)). Furthermore, co-transfection of TINCR-siRNA and TCPTP-siRNA remarkably reduced E-cadherin (epithelial marker) protein level and increased Vimentin (mesenchymal marker) protein level that were affected by TINCR silence in HCCLM3 HCC cells (Figure 3(g–i)). Additionally, the expression of TCPTP did not change in TINCR-silenced HCC cells (Supplementary Figure 2(a)). To further confirm that TINCR could compete with STAT3 in a competitive combination of TCPTP, the interaction of TCPTP and STAT3 was measured by co-Immunoprecipitation (co-IP) assay in TINCR-silenced HCC cells. The results showed that the interaction of TCPTP and STAT3 was increased in TCPTP-silenced HCC cells (Supplementary Figure 2(b)). Therefore, we concluded that TINCR inhibited the activity of TCPTP, thus regulating the phosphorylation of STAT3 and its downstream target genes.

### TINCR regulates HCC progression and STAT3 signaling via direct interacting with TCPTP

**Figure 4.**
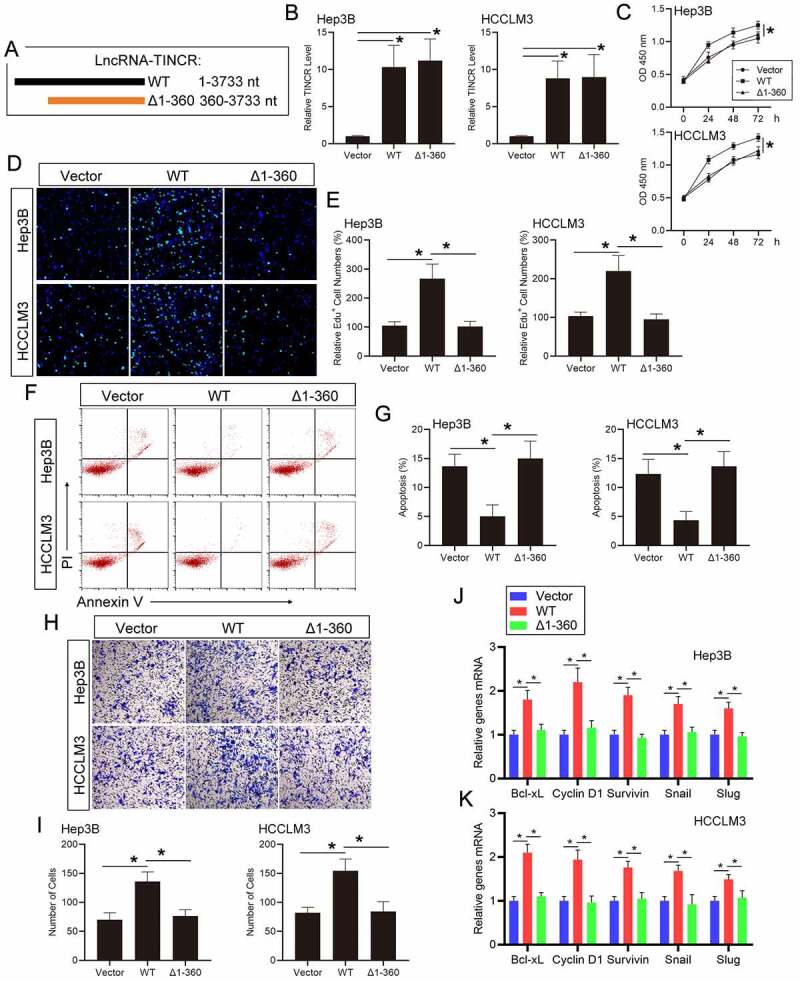
**LncRNA TINCR regulates HCC progression and STAT3 signaling via direct interacting with TCPTP**. (a) Schematic illustration of the plasmids encoding full-length (WT, 1–3733 nt) or the fragments (Δ1-360, 360–3733 nt) of TINCR. (b) Real-time PCR assay of TINCR expression in Hep3B and HCCLM3 cells transfected with WT or Δ1-360 TINCR. Data are given as mean ± SEM (n = 3, *P < 0.05, by ANOVA). The CCK-8 (c), EdU (d and e), apoptosis (f and g), and transwell (h and i) assays were conducted in Hep3B and HCCLM3 cells transfected with WT or Δ1-360 TINCR. Data are indicated as mean ± SEM (n = 3, *P < 0.05, by ANOVA). (j and k) Real-time PCR assay of STAT3 target genes expression in Hep3B and HCCLM3 cells transfected with WT or Δ1-360 TINCR. Data are given as mean ± SEM (n = 3, *P < 0.05, by ANOVA)

To further demonstrate that the function of TINCR on HCC cells is dependent on its direct interaction with TCPTP, we generated plasmids encoding full-length (WT, 1–3733 nt) or the fragments (Δ1-360, 360–3733 nt) of TINCR (Figure 4(a)). The real-time PCR assay showed that WT and Δ1-360 TINCR levels were remarkably increased following plasmids transfection in Hep3B and HCCLM3 HCC cells. (Figure 4(b)). The CCK-8 assay illustrated that WT TINCR overexpression promoted cell growth in Hep3B and HCCLM3 HCC cells. By contrast, Δ1-360 TINCR overexpression did not increase cell growth in contrast with the vector transfection cells (Figure 4(c)). Similarly, the results of EdU assay suggested that the ratio of EdU-positive cells was remarkably enhanced in Hep3B and HCCLM3 HCC cells with WT TINCR transfection but not with Δ1-360 TINCR transfection (Figure 4(d,e)). Then, the apoptosis assay showed that WT TINCR overexpression inhibited cell apoptosis in contrast with the control in Hep3B and HCCLM3 HCC cells. However, Δ1-360 TINCR overexpression did not affect cell apoptosis (Figure 4(f,g)). The above data illustrated that the influence of TINCR on HCC cell growth and apoptosis is dependent on its direct interaction with TCPTP. Then, the transwell assay showed that cell invasion capacity was remarkably enhanced with WT TINCR transfection, but not Δ1-360 TINCR transfection, in Hep3B and HCCLM3 HCC cells (Figure 4(h,i)). Additionally, the results of real-time PCR assay revealed that Cyclin D1, Bcl-xL, Survivin, Snail, and Slug mRNA levels were increased following WT TINCR overexpression in Hep3B and HCCLM3 HCC cells. These mRNA levels did not change in Δ1-360 TINCR overexpression in contrast with the vector transfection cells (Figure 4(j,k)). Moreover, the STAT3 phosphorylation level was increased in WT TINCR-transfected HCC cells, but not in Δ1-360 TINCR-transfected cells (Supplementary Figure 3(a)). To further confirm that TINCR could regulate TCPTP by inhibiting its catalytic activity, the STAT3 phosphorylation level was measured in TCPTP-silenced HCC cells. The results showed that the increasing of STAT3 phosphorylation level in TCPTP-silenced HCC cells did not reverse with TINCR overexpression (Supplementary Figure 3(b)). Therefore, the influences of TINCR on HCC cell infiltration and STAT3 target genes are dependent on its direct interaction with TCPTP.

## Discussion

LncRNA TINCR is upregulated and contributes to the poor prognosis of many cancers [25,26]. Most of studies suggest that TINCR promotes cancer progression by sponging microRNAs [27–29]. However, TINCR was firstly documented to bind to staufen1 (STAU1) protein and mediate differentiated mRNA stabilization [9]. Additionally, TINCR can interact with ATP-Citrate Lyase (ACLY), BRAF, and Staphylococcal Nuclease and Tudor Domain Containing 1 (SND1) in nasopharyngeal cancer, non-small cell lung cancer, and post-burn skin fibroblasts, respectively [30–32]. Therefore, the interaction proteins and molecular mechanisms of TINCR driving the cancer cell proliferation and metastasis contributing to HCC carcinogenesis have remained unresolved. Here, we employed RNA pull down and LC-MS/MS assays to determine TINCR-interacting proteins in HCC cells. At present, we found that TCPTP could be an important regulator of the influences of TINCR on HCC cell proliferation and metastasis. Besides, we use bioinformatics analysis (catRAPID omics algorithm) to investigate the binding regions of lncRNA TINCR and protein TCPTP complex. The predicted results suggested that the 1–360 nt region of TINCR might bind to aa 62–113 and aa 187–238 regions of TCPTP. Moreover, the structural analysis of the TINCR-TCPTP complex was simulated by using Protein Data Bank and mfold web server. Ultimately, we identified 1–360 nt region of TINCR, which directly bound with the phosphatase domain of TCPTP to inhibit its tyrosine phosphatase activity (Figures 1() and (3)).

Both STAT1 and STAT3 are dephosphorylated by TCPTP [19]. STAT3 is a specific substrate of TCPTP [18]. Moreover, the nuclear isoform of TCPTP (TC45) was reported to dephosphorylate STAT3 in the nucleus by interacting with STAT3 [18,33]. However, STAT3 play a key role in liver inflammation and cancer [34]. Additionally, previous studies have revealed that lncRNA TSLNC8 and DILC regulate liver cancer development via STAT3 signaling [35,36]. Herein, we primarily focused on the STAT3 as well as its downstream target genes. Firstly, we silenced TCPTP in TINCR-silenced HCC cells to investigate whether TINCR affected HCC carcinogenesis through TCPTP. The results showed that the increasing of cell growth, migration, infiltration and the reducing of apoptosis in TINCR-silenced HCC cells was remarkedly reversed with TCPTP silence. We know STAT3 signaling is involved in tumor cell proliferation, survival, infiltration, immunosuppression, obesity, stem cells as well as the pre-metastatic niche [37]. Moreover, the critical roles of activated STAT3 include cell apoptosis inhibition by up-regulating the expression of Bcl-xL, and Mcl-1, cell proliferation by up-regulating the transcription of cyclin D1 along with cyclin B1, and cell metastasis by regulating the expression of MMP2, MMP9, Snail, and Slug [37–40]. Secondly, we generated encoding the fragments (Δ1-360) of TINCR plasmids to further confirm that the influences of TINCR on HCC cells was dependent on its direct interaction with TCPTP. Then, the results showed that WT TINCR overexpression promoted cell proliferation, metastasis, and STAT3 target genes expression in HCC cells. However, Δ1-360 TINCR overexpression did not affect these cellular functions.

## Conclusion

Taken together, TINCR directly bound to TCPTP and suppressed the dephosphorylation of STAT3, thus promoting STAT3 activation and its downstream target genes in HCC progression and tumorigenicity. We document herein a new mechanism underlying HCC cell proliferation and metastasis through the interaction of TINCR with TCPTP and activation of STAT3 target genes. Our data may provide a strategy for using TINCR as a potential biomarker and a therapeutic target for HCC.

## Supplementary Material

Supplemental Material
