# Supplementary material for: Long non-coding RNA TINCR promotes hepatocellular carcinoma proliferation and invasion via STAT3 signaling by direct interacting with T-cell protein tyrosine phosphatase (TCPTP)
Source: Bioengineered. 2021 May 30;12(1):2119–31. doi: 10.1080/21655979.2021.1930336 (PMC8806792; doi:10.1080/21655979.2021.1930336)
Supplement: Supplemental Material [file KBIE_A_1930336_SM7717.zip › supplementary/Supplementary Figures.docx]

**Supplementary figures**


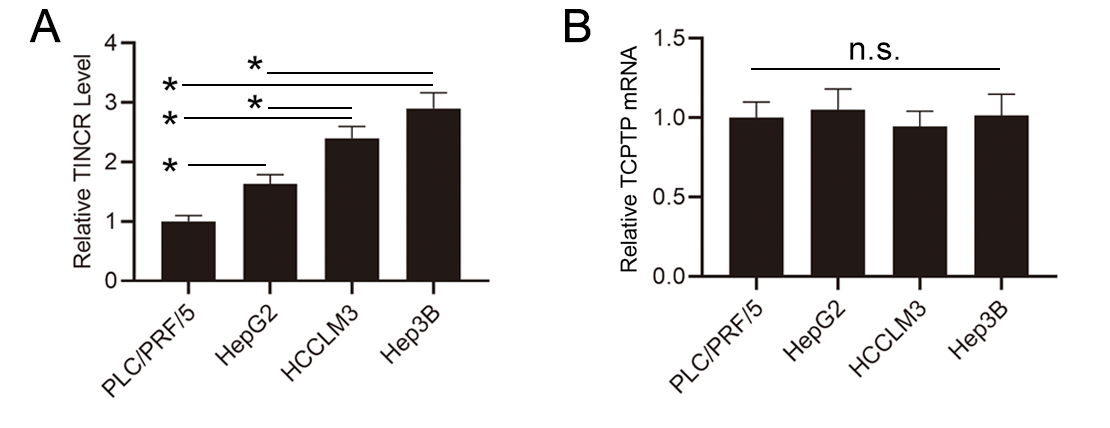


**Supplementary Figure 1. The levels of LncRNA TINCR and TCPTP in HCC cell lines. (A)** Real-time PCR assay of TINCR expression in HCC cell lines. Data are indicated as mean ± SEM (n = 3, **P* < 0.05, by ANOVA). **(B)** Real-time PCR assay of TCPTP expression in HCC cell lines. Data are indicated as mean ± SEM (n = 3, n.s., no difference).


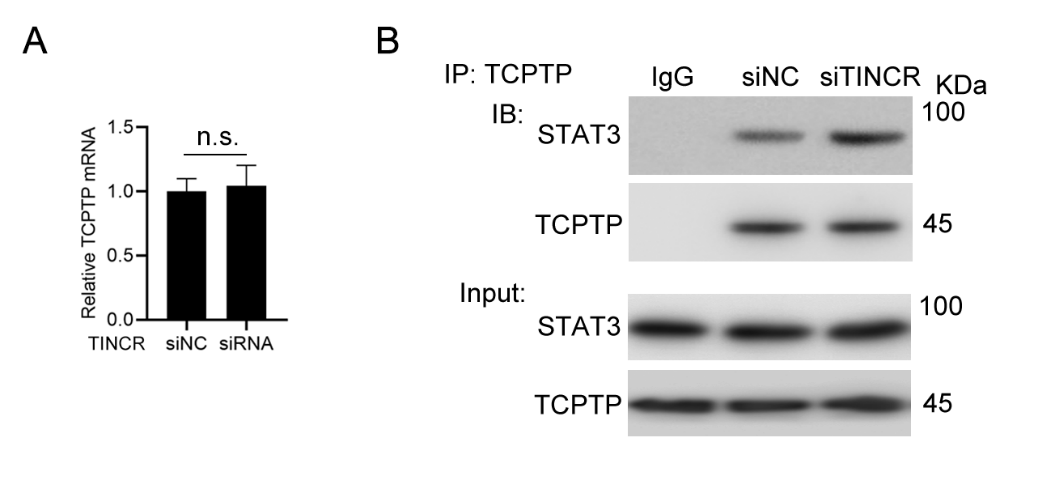


**Supplementary Figure 2. The knockdown of TINCR increased the interaction of STAT3 and TCPTP in HCC cells. (A)** Real-time PCR assay of TCPTP expression in TINCR-silenced HCCLM3 cells. Data are indicated as mean ± SEM (n = 3, n.s., no difference). **(B)** The association between STAT3 and TCPTP protein was shown in co-Immunoprecipitation (co-IP) assays after silencing TINCR in HCCLM3 cells. IP was performed with anti-TCPTP antibody and immunoblot (IB) was performed using anti-STAT3 antibody. IgG group was used as negative control. Input controls of STAT3 and TCPTP were performed on total cell protein lysates. Data shown represent three independent experiments.


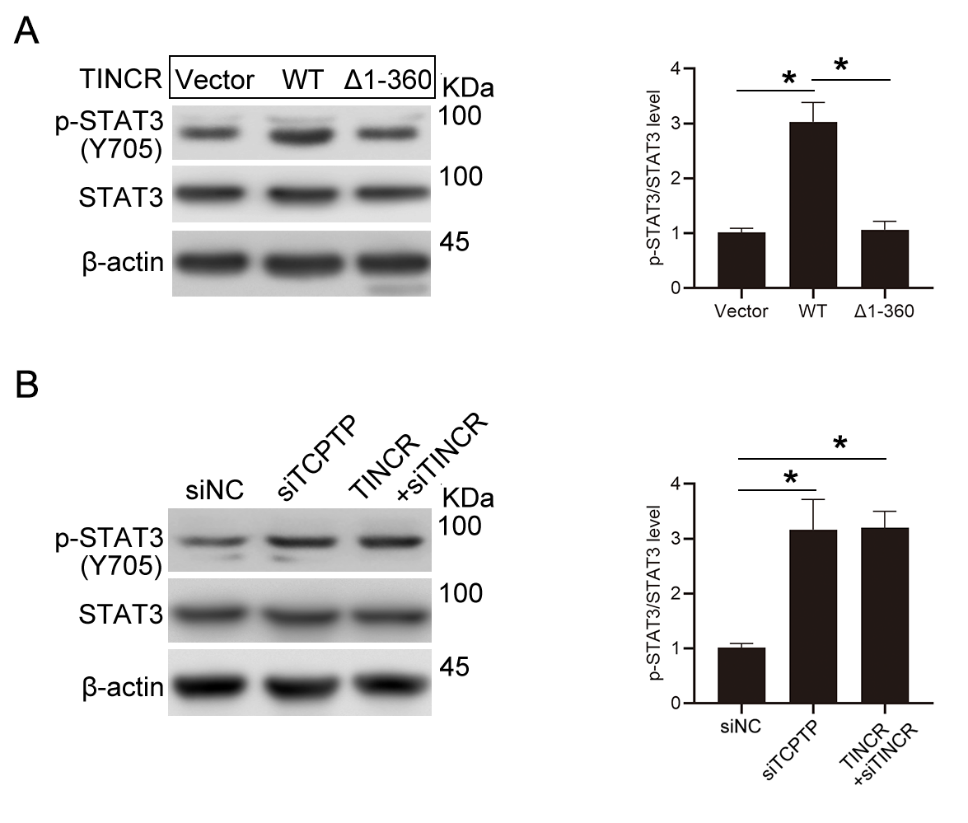


**Figure 3. LncRNA TINCR inhibited TCPTP catalytic activity on STAT3.** **(A)** Western blot assay of STAT3 phosphorylation in HCCLM3 cells transfected with WT or Δ1-360 TINCR. Data are given as mean ± SEM (n = 3, **P* < 0.05, by ANOVA). **(B)** Western blot assay of STAT3 phosphorylation in HCCLM3 cells transfected with siRNA-NC, siRNA-TCPTP, or co-transfected with siRNA-TCPTP and WT TINCR. Data are indicated as mean ± SEM (n = 3, **P* < 0.05, by ANOVA).
