# Supplementary material for: Long non-coding RNA TINCR promotes hepatocellular carcinoma proliferation and invasion via STAT3 signaling by direct interacting with T-cell protein tyrosine phosphatase (TCPTP)
Source: Bioengineered. 2021 May 30;12(1):2119–31. doi: 10.1080/21655979.2021.1930336 (PMC8806792; doi:10.1080/21655979.2021.1930336)
Supplement: Supplemental Material [file KBIE_A_1930336_SM7717.zip › supplementary/Supplementary Table 1.docx]

**Table 1 Primers and siRNAs used in this study.**

| Primers and siRNAs | Sequence (5’-3’) |
| --- | --- |
| As-f | TAATACGACTCACTATAGGG TTGTTTTCAAACATGTAATC |
| As-r | GGGCGGGCGGAGCGCGGGCG |
| FL-f | TAATACGACTCACTATAGGGGGGCGGGCGGAGCGCGGGCG |
| D1-f | TAATACGACTCACTATAGGG AGCGACCCCAGGTAGTCTGG |
| D2-f | TAATACGACTCACTATAGGG AGGCCTCCAACTGTGCCCCA |
| D3-f | TAATACGACTCACTATAGGG GCTTTGCAGAATGACTTGGG |
| R1 | TTGTTTTCAAACATGTAATC |
| D4-f | TAATACGACTCACTATAGGGGGGCGGGCGGAGCGCGGGCG |
| D4-r | CAGCTCCAGCAGGTCTGCCT |
| RIP-f | CTGCTACCGCTGACCGTG |
| RIP-r | GCCGCGCGTTGTAGTAGAAG |
| TINCR-f | CCAAGGAGGTTGTCAGGGAC |
| TINCR-r | TAGATACACGCATGTGGCCC |
| TCPTP-f | GAAGAGTTGGATACTCAGCGTC |
| TCPTP-r | TGCAGTTTAACACGACTGTGAT |
| Bcl-xL-f | GAGCTGGTGGTTGACTTTCTC |
| Bcl-xL-r | TCCATCTCCGATTCAGTCCCT |
| Cyclin D1-f | GCTGCGAAGTGGAAACCATC |
| Cyclin D1-r | CCTCCTTCTGCACACATTTGAA |
| Survivin-f | AGGACCACCGCATCTCTACAT |
| Survivin-r | AAGTCTGGCTCGTTCTCAGTG |
| Snail-f | TCGGAAGCCTAACTACAGCGA |
| Snail-r | AGATGAGCATTGGCAGCGAG |
| Slug-f | TGTGACAAGGAATATGTGAGCC |
| Slug-r | TGAGCCCTCAGATTTGACCTG |
| β-actin-f | CATGTACGTTGCTATCCAGGC |
| β-actin-r | CTCCTTAATGTCACGCACGAT |
| TINCR siRNA-1 | 1523-GAAATAATGTTTTAGTTAAGA |
| TINCR siRNA-2 | 2160-GAAAATGGGGCATTTAATAAT |
| TINCR siRNA-3 | 3680-CAGAAATGCTGTTTTGAGAGT |
